# The Ontogeny and Function of Placental Macrophages

**DOI:** 10.3389/fimmu.2021.771054

**Published:** 2021-10-21

**Authors:** Jake R. Thomas, Praveena Naidu, Anna Appios, Naomi McGovern

**Affiliations:** Department of Pathology and Centre for Trophoblast Research, University of Cambridge, Cambridge, United Kingdom

**Keywords:** macrophage, placenta, pregnancy, ontogeny, development

## Abstract

The placenta is a fetal-derived organ whose function is crucial for both maternal and fetal health. The human placenta contains a population of fetal macrophages termed Hofbauer cells. These macrophages play diverse roles, aiding in placental development, function and defence. The outer layer of the human placenta is formed by syncytiotrophoblast cells, that fuse to form the syncytium. Adhered to the syncytium at sites of damage, on the maternal side of the placenta, is a population of macrophages termed placenta associated maternal macrophages (PAMM1a). Here we discuss recent developments that have led to renewed insight into our understanding of the ontogeny, phenotype and function of placental macrophages. Finally, we discuss how the application of new technologies within placental research are helping us to further understand these cells.

## Introduction

**Figure 1:**
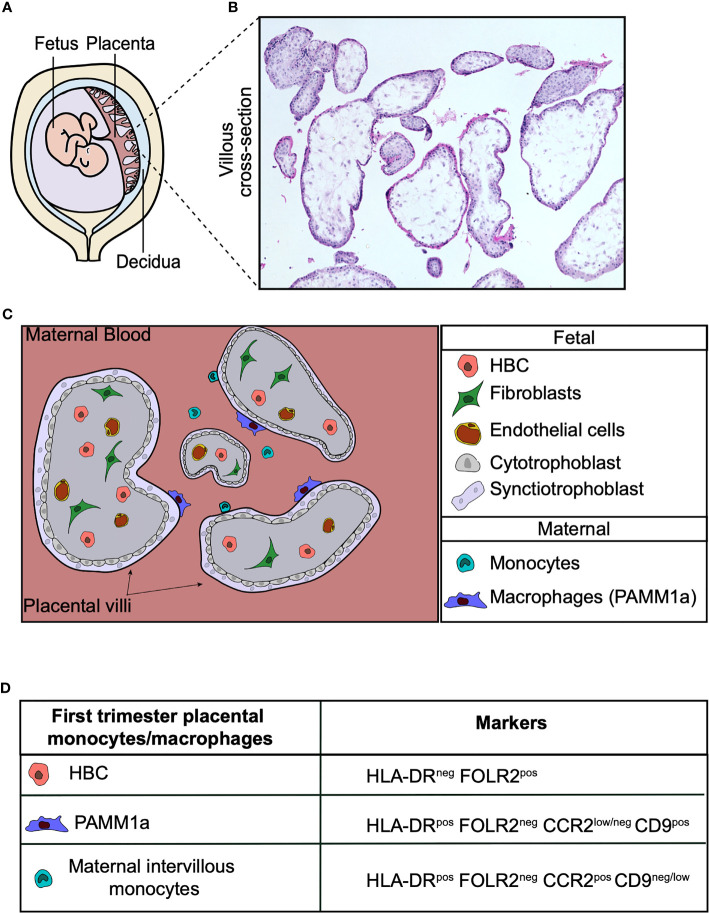
Human placental macrophages. **(A)** Illustration of the human placenta. **(B)** Hematoxylin and eosin stain of first trimester placental villi. **(C)** Cross-section diagram of first trimester placental villi indicating the localization of placental macrophages. **(D)** Surface markers of monocyte and macrophage populations found in first trimester placental digests. Hofbauer cells (HBC), PAMM1a (placental associated maternal macrophages).

The placenta is the first and largest organ the fetus makes. It is the interface between the mother and fetus, and a normal functioning placenta is crucial for successful pregnancy. The placenta carries out a range of functions, including mediating the exchange of gases, nutrients and waste between the fetus and mother. It is also a highly efficient barrier, preventing the transfer of many harmful pathogens to the fetus. Hofbauer cells (HBC) are a population of tissue-resident macrophages found within human placental villi. These cells appear very early during development and have been identified at day 18 post-conception (1, 2). HBC are the only significant immune cell population found within the normal healthy human placenta. In addition to fetally-derived HBC, a population of placenta associated maternal macrophages (PAMM1a) have recently been characterized (3) that can be found adhered to the surface of placental villi (**Figure 1**).

The properties of macrophages are determined by local physical and trophic signalling cues in their given tissue niche, resulting in the expression of specialized transcriptional programs (4, 5) and functional properties. Accordingly, both HBC and PAMM are thought to play niche-specific roles in order to promote normal placental function and development.

A select group of pathogens are capable of crossing the placenta and causing congenital disease. These pathogens are referred to as TORCH: Toxoplasma gondii, Other[(HIV, *Listeria monocytogenes*, Candida Albicans, varicella zoster virus, amongst others including new emerging pathogens such as the Zika virus (ZIKV)], Rubella, Cytomegalovirus and Herpes simplex viruses. When maternal infection with a TORCH agent occurs during pregnancy the transplacental infections rates are typically low, for example *in utero* HIV and CMV transmission rates are 7% (6, 7) and 0.5-2% (8) respectively. The limited repertoire of pathogens capable of transplacental infection and their low transmission rates suggest that the placenta has multiple mechanisms in place to prevent infection. As the only immune cells found within the placental villi, HBC are likely to have crucial functions in the prevention of transplacental infections. PAMM1a also may act to prevent microbe transmission at sites of damage to the syncytium, in addition they could provide a route of infection for microbes that survive and replicate in macrophages. However, these roles have not yet been fully explored, and it is unclear as to why HBC and PAMM1a are capable of preventing the transmission of some pathogens but are permissive to others.

In this review we will discuss the ontogeny, phenotype and properties of both HBC and PAMM1a, consider their roles in homeostasis to promote normal placental function throughout gestation, and their contributions to the defense against, or susceptibility to a range of pathogens. Finally, we will discuss resources and experimental models that are available for the further study of HBC and PAMM1a.

## HBC

### The Phenotype of First Trimester Hofbauer Cells

The villous core of the human placenta consists of connective cells embedded within an extracellular matrix. Mesenchymal cells, or undifferentiated stromal cells, are the principal cell type until the end of the second month of gestation, with fibroblasts starting to appear from approximately the third month of gestation (9). The long thin cytoplasmic processes of first trimester mesenchymal cells connect with neighboring cells to form a series of stromal channels (9, 10). These channels are relatively large, 20-50 μm in diameter, and are thought to aid in the diffusion of nutrients through the stroma. Within these first trimester stromal channels, HBC can be found. Their pleomorphic morphology reflects their dynamic migratory properties, where electron microscopy imaging of first trimester placenta have captured HBC migrating from one channel to the next, to the steady-state (11).

Phenotypically, HBC have been characterized as CD14^+^ CD68^+^ cells that express a variety of macrophage markers including scavenger receptor CD163, mannose receptor CD206, Fc receptor CD64 and folate receptor 2 (FOLR2) (3). Historically, microscopy analysis has demonstrated that HLA-DR is not expressed in the first trimester villi (12). However, analysis of placental digests yielded macrophages that are heterogenous for HLA-DR (13), leading to confusion regarding the true phenotype of HBC. A recent study using HLA allotype antibodies to accurately distinguish fetal and maternal cells from placental digests revealed that first trimester HBC do not express HLA-DR. CD14^+^ cells expressing HLA-DR from first trimester digests were found to be maternal in origin (3). The lack of HLA-DR expression by first trimester HBC is unusual, as it is typically described as a canonical marker of human macrophage identity and its expression is reliably observed in adult and 2^nd^ trimester fetal macrophages across tissues (14). This could be attributed to their ontogeny (discussed below) or to the unique environment of the first trimester placenta, where T cell populations are not found in the steady-state.

### First Trimester HBC Ontogeny

The first wave of embryonic hematopoiesis is called primitive hematopoiesis and in the mouse it occurs solely in the yolk sac. Primitive hematopoiesis gives rise to erythrocytes, megakaryocytes and macrophages. These macrophages are commonly termed primitive macrophages and are distinct to those generated through definitive hematopoiesis as they are generated independently of monocytes. That is primitive macrophages arise directly from primitive HSCs, also known as erythro-myeloid progenitors (15). Murine fate-mapping models have demonstrated that yolk sac derived primitive macrophages rapidly seed all embryonic tissues and are crucial for embryonic development (15). When definitive hematopoietic stem cells emerge subsequently in different anatomical sites, such as the aorta-gonad mesonephros (AGM), fetal liver and finally the bone marrow, monocytes are generated that can enter tissues to differentiate into macrophages (16–18). Hence, by the end of gestation the ontogeny of macrophages across tissues display variable contributions from primitive and definitive hematopoietic precursors, as has been extensively discussed elsewhere (15).

As HBC have been observed from day 18 post-conception, it is predicted that first trimester HBC are derived from primitive HSCs, as definitive hematopoiesis has not begun at this point of gestation (19, 20). This is supported by analysis of scRNAseq data which demonstrated that first trimester HBC are a homogenous population, and fetal monocytes are not found in first trimester placenta data sets (3). Additionally, HBC and primitive yolk sac macrophages have highly correlated gene expression profiles and phenotypes, both expressing FOLR2 and lacking HLA-DR (3).

The origin of human HBC however remains unresolved. There are three potential sources of origin of first trimester HBC: 1) HBC are generated in the yolk sac and migrate to the placenta, 2) HBC arise from precursors within the placenta, 3) a combination of both. Unfortunately, murine studies cannot help resolve this question as discussed further below. However, a combination of techniques including immunohistochemistry (21), analysis of somatic mutation acquisition using whole-genome sequencing (22) and colony forming assays (21), have helped elucidate the origin of human HBC. Studies using these techniques have demonstrated that both the human placenta (22) and yolk sac (23) arise from the extra-embryonic mesoderm, which in turn is derived from the hypoblast. Macrophages have been found to appear simultaneously within both organs at 16-18 days post conception (pp.). Finally, putative macrophage precursors in the pre-circulation placenta have been identified (21). These factors combined strongly suggests that HBC are generated *de novo* in human placental villi.

### The Functional Properties of First Trimester HBC

**Figure 2:**
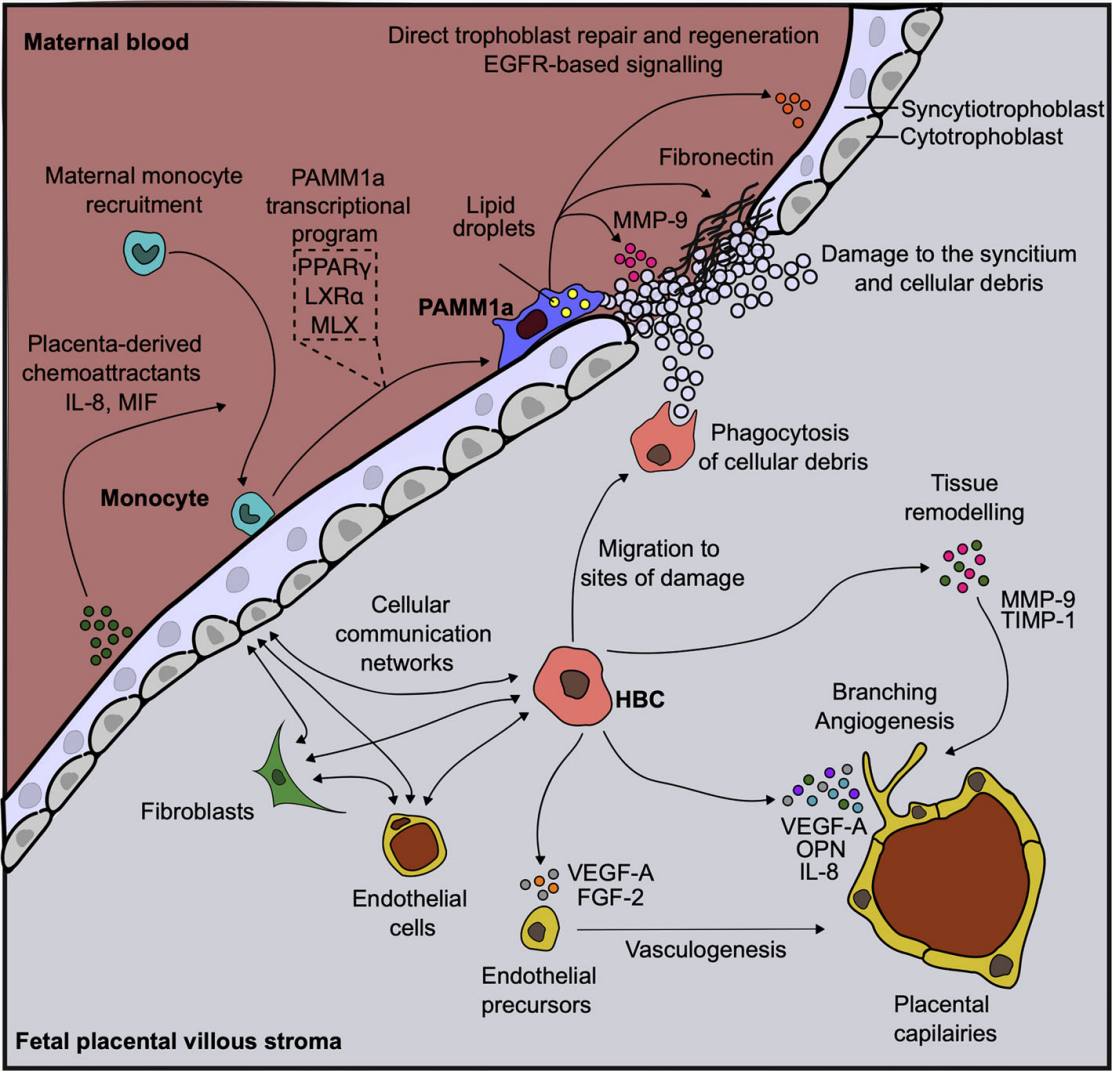
Human placental macrophages have diverse functional properties. Diagram demonstrating the diverse roles that placenta associated maternal macrophages (PAMM1a) and Hofbauer cells (HBC) are thought to play in the steady-state. Vasculature endothelial growth factor, (VEGF); fibroblast growth factor, (FGF); osteopontin, (OPN); matrix metalloprotease, (MMP); tissue inhibitor of metalloproteinase, (TIMP); Max-like protein X, (MLX); liver X receptor, (LXR); peroxisome proliferator-activated receptor, (PPAR); endothelial growth factor receptor, (EGFR); macrophage migration inhibitory factor, (MIF); interleukin, (IL).

The functional properties of HBC have been the subject of great interest as they are the only immune cells found within the stromal core of first trimester placenta and are likely to display diverse roles (**Figure 2**). Through their close association with endothelial progenitors and primitive vessels (24), and secretion of factors such as vascular endothelial growth factor (VEGF) (3, 25), sprouty proteins (26) and osteopontin (3), HBC are thought to aid in early placental vasculogenesis and angiogenesis, as well as regulating branching morphogenesis of the villous tree. HBC also secrete tissue inhibitor of metalloproteinase (TIMP-1) and matrix metalloproteinase (MMP-9), factors involved in remodeling of placental vessels (27, 28). A greater understanding of the interaction potential of HBC with other placental cells can be gained by combining HBC protein secretion data with scRNAseq gene expression data for cognate receptors. This analysis reveals that placental endothelial cells, through the expression of kinase insert domain receptor (KDR) and neuropilin 1 (NRP1) are the main target of HBC secreted VEGF-A. In addition, endothelial cell expression of CD44 and integrin complexes make them the likely responders to osteopontin (OPN) secreted by HBC (3). Indeed these interactions have been shown to be important for the endothelial biology and angiogenesis (29, 30). Additionally, HBC are predicted to signal to placental fibroblasts *via* IL-6, and to villous cytotrophoblast *via* both OPN and granulocyte-macrophage colony-stimulating factor (GM-CSF) (3). Hence it can be seen through a range of factors they secrete that HBC mediate the biology of other placental cell types, and are therefore likely to play a critical role in promoting and regulating placental vascularization and growth.

HBC are also thought to aid in placental development through the efficient clearance of debris, a process known as efferocytosis, as the organ undergoes rapid growth. This is illustrated through their high expression of a range of scavenger receptors including CD163, CD68, AXL and TIM-1 (3, 31). AXL is a member of the TAM (Tyro3, Axl and Mertk) receptor tyrosine kinase family that recognizes phosphotidylserine (PtdSer) on the surface of apoptotic cells. TAM receptors are important as they inhibit inflammation during apoptotic cell efferocytosis *via* a negative feedback loop involving activation of suppressor of cytokine signaling-1 and -3 that inhibit cytokine and Toll-like receptor (TLR) signalling pathways (32). In line with their high expression of phagocytic receptors, HBC display elevated phagocytic capacity in comparison with PAMM1a (3). In addition to the clearance of debris, the enhanced phagocytic capacity of HBC is also likely to be important for the clearance of harmful molecules that can enter the placenta, such as immune-complexes and black carbon particles (combustion-derived particulate matter) (33).

The demonstration that HBC cluster at sites of fibrinoid necrosis *in vivo* (12) and also to sites of villous damage *in vitro* (34), indicates that the migratory capacity of first trimester HBC is important for placental function, repair and defense. TGFβ1 was found to be highly expressed at sites of tissue injury and recruited HBC, suggesting it is involved in the placental wound repair process (34). Hence, it can be seen that HBC are migratory cells that are well equipped for the effective clearance of apoptotic cells and potentially harmful molecules that may enter the placenta without triggering inflammation, key processes for the maintenance of homeostasis within the villous stroma.

### The Impact Of The Changing Needs of the Placenta on HBC Properties

The human placenta is a highly dynamic organ throughout pregnancy, growing until birth and meeting the changing needs of the rapidly developing fetus. By full-term the villous cytotrophoblast layer becomes discontinuous and covers only 25% of the villous surface, whereby only a thin syncytial layer separates most of the villous core from maternal blood (9). The loose, open, stromal channel structures that are observed in the first trimester placenta are replaced by a more compact and denser stroma, with the placental blood vessels growing to take up the majority of space within the villi. It is unclear as to how these changes in the placental microenvironment impact on HBC properties, as relatively few studies have compared first trimester with full-term HBC.

When definitive hematopoiesis begins in other anatomical sites, it has been proposed that other immune cells, such as dendritic cells, may enter the placental villi. Fetal blood flow to the placenta becomes fully established from the 10th week of gestation, which could permit the influx of dendritic cells from the fetal circulation. However, very low numbers of dendritic cells have been identified in placental scRNAseq datasets (3, 35) and these cells in first trimester samples are likely derived from maternal blood contamination, as indicated by the expression of X-chromosome specific *XIST* in male fetal donor samples (3). Convincing localization data has not been provided to demonstrate dendritic cells leave the fetal blood to enter the placental villi at later time points during gestation. Given this, the rarity of T cells (36) and the lack of lymphatic vessels in the placenta, it is unlikely that dendritic cells play a role in placental function in health.

In contrast, fetal blood monocytes are thought to enter the placental villi when definitive hematopoiesis begins. HBC have been shown to upregulate HLA-DR expression by full-term (12). The elevated expression of HLA-DR by HBC may be due to fetal blood monocyte-derived macrophages appearing in the placenta and replacing the initial population of HBC derived from primitive hematopoiesis; however this remains unclear. Further changes that HBC undergo during gestation and how these changes aid in placental function remain undefined.

### The Role of HBC in Transplacental Infection

As HBC are the only immune cells located within the placental villi, they are expected to play a major role in helping to defend the fetus from infection, should a microbe cross the outer syncytium layer. A shared characteristic of many TORCH agents is the ability to survive and replicate in macrophages. Given this, it is surprising that there are relatively few studies that have analysed the interaction of HBC with microbes and sought to understand their role in transplacental infection. HBC must strike a balance between adequately protecting the placenta from infection and generating potentially damaging inflammatory responses, which have been implicated in causing miscarriages (37). HBC are often described as tolerogenic cells, however, the response they initiate is highly dependent on signalling cues. For example, *in vitro* assays have demonstrated that HBC secrete pro-inflammatory cytokines in response to toll-like receptor (TLR) stimulation. In comparison with PAMM1a, HBC have a potent response to TLR-6 stimulation, reflective of their high expression of this receptor, secreting high amounts of pro-inflammatory mediators such as GM-CSF, IL-6, IL-8 and CCL-3. HBC have potent microbicidal effector functions, with the capacity to produce high amounts of reactive oxygen species and anti-microbial enzymes such as cathepsin B (3). In addition, the containment of microbes by HBC in tetraspanin-positive compartments that are accessible to neutralizing maternal-derived antibodies, is thought to be important in preventing the transmission of microbes to the fetal blood stream (38).

Of all the TORCH agents, the interaction of HBC with HIV has been studied to the greatest extent. HBC express the HIV entry receptors CD4 (39), CCR5, CXCR4 and DC-SIGN (7) and are susceptible to HIV infection. During pregnancy the chance of HIV crossing the placenta and infecting the fetus, when the mother has no protective antiretroviral therapy, is ~20% (40). It has been proposed that the unique properties of HBC play an important role in sequestrating and neutralizing HIV. For example, *in vitro* assays have demonstrated that HBC can limit HIV-1 replication by induction of immunoregulatory cytokines such as IL-10 (7). Also, the sequestration of HIV in acidic compartments by HBC aids in HIV neutralization (38), as HIV is sensitive to low pH and proteases (41). Cases of HIV infected placenta are not associated with inflammation of placenta, termed villitis, indicating that HBC act to regulate placental HIV infection without triggering a pro-inflammatory response which could be detrimental to the pregnancy (42).

The response of HBC towards Zika virus has also been studied. Zika virus (ZIKV) is an arbovirus of the *Flavivirus* genus. Few cases of ZIKV infections were reported in humans before 2007. However, this changed with the outbreaks in Micronesia, French Polynesia and Brazil and the Americas from 2007 - 2015. In these naive populations congenital ZIKV infection, especially during early pregnancy, caused a variable syndrome of severe malformations in the fetus, termed congenital Zika syndrome (CZS), that can include microcephaly at delivery or postnatally, reduction in cerebral volume, ventriculomegaly, subcortical calcifications, ocular defects and neuro-muscular abnormalities (43). HBC highly express the ZIKV entry receptors AXL and TIM1 (3, 44). A combination of *ex vivo* (44) and *in vitro* (45) assays have demonstrated that HBC can be infected with ZIKV and support its replication. Once infected, HBC may then disseminate the virus to fetal blood vessels. ZIKV-infected placentas exhibit hyperplasia of HBC, potentially amplifying virus production by these cells in the villous core, and lack classical signs of inflammation, necrosis or scarring in the placenta. This is striking considering that the virus can cause necroinflammatory reactions when it reaches the fetal brain. This suggests that ZIKV has an ability to evade a pro-inflammatory response that is specific to the placenta (42). In contrast to these studies, HBC isolated from full-term placenta (>37 weeks GA) infected with ZIKV *in vitro* do adopt a mildly activated phenotype, increasing their expression of activation markers CD80 and CD86 and secretion of pro-inflammatory mediators IFNα, IL-6, MCP-1 and IP-10 (45). The differences in findings between these studies suggest that signalling cues specific to the placental niche may act to prevent HBC from adopting a pro-inflammatory phenotype in response to ZIKV infection, and these are lost during *in vitro* assays. Hence, it is of interest to further explore how other placental cells, such as trophoblast cells and fibroblasts regulate HBC biology.

Further developing our understanding of the interaction of HBC with infectious microbes will help us to understand how certain pathogens, such as cytomegalovirus, cause placental malfunction, while others do not.

## PAMM

### Diversity and Phenotype of First Trimester PAMM

Maternal leukocytes were first observed on the surface of the placenta by electron microscopy (46, 47), however the phenotype and properties of these cells remained unexplored until recently. Placenta-associated maternal monocytes/macrophages (PAMM) adherent to the placental surface were first characterized in-depth using anti-HLA allotype antibodies in flow cytometric panels and female-specific genes, such as *XIST*, in scRNAseq datasets derived from male fetus placental digests (3). Further characterization of PAMM by flow cytometry, led to the development of a flow cytometric gating strategy that allowed the distinction of PAMM subsets found in the intervillous space. These maternal subests are HLA-DR^+^FOLR2^-^CD9^-/int^CCR2^+^ monocytes and a population of HLA-DR^+^FOLR2^-^CD9^+^CCR2^int/-^ macrophages termed PAMM1a. The PAMM subsets were consistently found in first trimester placental digests (7 – 11^th^ week of gestation) (3). While PAMM1a-like cells have been observed on full-term placental villi (46), they have yet to be fully characterized.

### PAMM Recruitment and Differentiation

As the placenta is a transient organ, PAMM1a must be derived from maternal blood monocytes that are found in the intervillous space, ultimately originating from the bone marrow. From the 10^th^ week of gestation maternal blood fills the intervillous space due to maternal spiral artery remodeling (9), providing a source of monocytes that could in turn differentiate into PAMM1a. However, PAMM1a have been observed in placental digests from as early as 7wk EGA, before this process becomes fully established. The early appearance of PAMM1a may be due to a low level of maternal blood flow to the intervillous space prior to the 10^th^ week of gestation or due to monocytes migrating from the decidua, which has been shown to be enriched with monocytes during the first trimester of pregnancy (35, 48). Explant culture assays have revealed that placental villi constitutively secrete a diverse range of cytokines and chemokines (49, 50). Macrophage migration inhibitory factor (MIF) is amongst the most highly expressed cytokine in both of these studies, which has been shown to be a potent chemoattractant of monocytes (51–53). Although the secretion of MIF could be an artefact of the non-physiological conditions of explant cultures, it has been widely reported as a factor highly expressed in the first trimester of human pregnancy (54, 55).

Once monocytes adhere to the placental surface they can differentiate into PAMM1a (macrophages). scRNAseq analysis revealed a continuous transcriptomic differentiation trajectory from intervillous maternal monocytes to PAMM1a, resulting in the upregulation of a transcriptional program and phenotype specific to the placental surface (3). The precise signalling cues from the placenta that govern this process are yet to be fully elucidated. Notably, the syncytiotrophoblast which forms the outer layer of placental villi have been reported to secrete M-CSF (49) a critical mediator of the monocyte-to-macrophage transition.

### The Functional Properties of First Trimester PAMM

The observation that PAMM1a are embedded onto the synctium of placentas from healthy pregnancies suggests that these cells have important roles in healthy placental function, including the repair and development of the placenta (**Figure 2**). The syncytium always contains sites of damage and fibrin deposition during healthy pregnancy (47). This poses a significant risk to the fetus during pregnancy, as the syncytium forms a highly effective physical barrier to infection and breaks in its surface may permit the passage of opportunistic infections from mother to child. PAMM1a were found to be localized to sites of damage on the surface of the first trimester placenta and were found to secrete matrix metalloprotease (MMP)-9 and fibronectin, both critical regulators of tissue repair. This suggests that PAMM1a play a role in the maintenance and repair of the placenta during healthy pregnancy. Furthermore, PAMM1a are loaded with lipid droplets (3) and highly express the transcription factors peroxisome proliferator-activated receptor (PPAR)γ and liver X receptor (LXR)α that are associated with lipid metabolism and storage [determined through analysis of whole-genome sequencing data, deposited at ArrayExpress E-MTAB-6701 (35)]. Both of these are hallmarks of macrophages that are engulfing cellular debris and apoptotic cells *via* phagocytosis (56–58). Cell-cell communication network analysis also revealed that PAMM1a might signal to villous cytotrophoblast and syncytiotrophoblast in an EGFR-dependent fashion, through the secretion of amphiregulin (AREG), epiregulin (EREG) and heparin-binding EGF-like growth factor (HBEGF) [determined through analysis of whole-genome sequencing data, deposited at ArrayExpress E-MTAB-6701 (35)]. These factors are known to be important in driving trophoblast proliferation and differentiation (59–64). Therefore, PAMM1a are likely driving both the repair and regeneration of the placental surface in the first trimester of human pregnancy.

Interestingly, the transcriptional program upregulated in PAMM1a upon differentiation showed significant overlap with gene signatures from other recently described macrophages in various disease states, including adipose tissue during obesity (65), the liver during metabolic-associated fatty liver disease (MAFLD) (66, 67) and cirrhotic fibrosis (68), and atherosclerotic plaques (69). All of these populations are locally derived from monocytes upon the onset of disease, and their presence across tissues suggest a conserved macrophage transcriptional program in response to these fatty or scar-tissue related diseases, including the following genes; *SPP1, FABP5, TREM2, APOC1, GPNMB, LGALS3, CD9, LPL, LIPA, APOE, LGALS1, LSP1, PLIN2, SDS, MATK, PPARG, NR1H3*. Despite adopting this conserved transcriptional program, PAMM1a are unique among this group of macrophages as they are the only ones to arise in a healthy tissue. This has interesting implications for our understanding of macrophages in these states, as some of the features that are negatively attributed with disease, are actually important for tissue repair and function. Hence, PAMM1a provide valuable insight into the mechanisms that macrophages use to repair tissues in health and the steady-state. Further comparison of PAMM1a with macrophages found in diseased tissues will aid in the development of our understanding of how repair processes can, in certain circumstances, lead to disease.

### The Role of PAMM in Transplacental Infection and Intervillositis

The localization of PAMM1a at sites of damage on the syncytium makes them ideal candidates for the defense of the placenta against infections. In line with this, PAMM1a have been shown to respond potently to TLR stimulation (3). The specificity of responses to inflammatory challenges by PAMM1a is complementary and non-redundant with those of HBC. HBC were found to be highly responsive to TLR6 stimulation, but not TLR7 stimulation, but the inverse was found for PAMM1a. This suggests that HBC and PAMM might act cooperatively to defend the placenta from bacteria and single-stranded RNA viruses.

The activation of PAMM1a however, can also potentially contribute to disease. For example, inflammation of the intervillous space, known as intervillositis, is defined as a diffuse infiltration of mononuclear cells (lymphocytes and monocytes) of maternal origin into the intervillous space of the placenta. This can result in intrauterine growth restriction which can lead to miscarriage or stillbirth. Maternal infection is the most common cause of intervillositis, although cases of unknown etiology have also been described (70). Intervillositis is commonly seen in malaria infections, where increased fibrin deposition and prominent syncytial knots are frequently observed. Maternal monocytes and macrophages are the most abundant population of the inflammatory infiltrate and may prolong inflammation in the intervillous space, negatively impacting on pregnancy (71). The properties of trophoblast cells also change in intervillositis, such as the upregulation of intercellular adhesion molecule (ICAM) expression (72), which could in turn lead to increased PAMM1a adhesion through lymphocyte function-associated antigen (LFA)-1 expression.

PAMM1a may also provide opportunistic pathogens with a mode of entry into the placenta. Syncytiotrophoblast cells are resistant to infection with many TORCH agents and it remains unclear as to how various microbes, such as HIV, cross the syncytium to infect the placenta. It has been proposed that infected circulating leukocytes may adhere and fuse to the syncytium, resulting in a route of pathogen transmission. This may occur through syncytin, the envelope glycoprotein of human endogenous retrovirus family W1 expressed by trophoblast cells, and the syncytin receptor ASCT2, that is expressed by some immune cells, such as T cells. It was recently found that HIV infected T cells, fuse with trophoblast cells and thereby transmit the virus to trophoblast cells (73). While it remains unclear as to whether PAMM1a express ASCT2, given that they are known to interact with syncytiotrophoblast cells it can be expected that if infected PAMM1a cells adhere to the placenta they can also contribute to transplacental infection.

Hence, it can be seen that while PAMM1a play an important role in mediating placental biology in health, they may also contribute to disease by driving inflammation and providing a route of entry for microbes.

## Challenges and Experimental Models for the Future Study of Placental Macrophages

**Figure 3:**
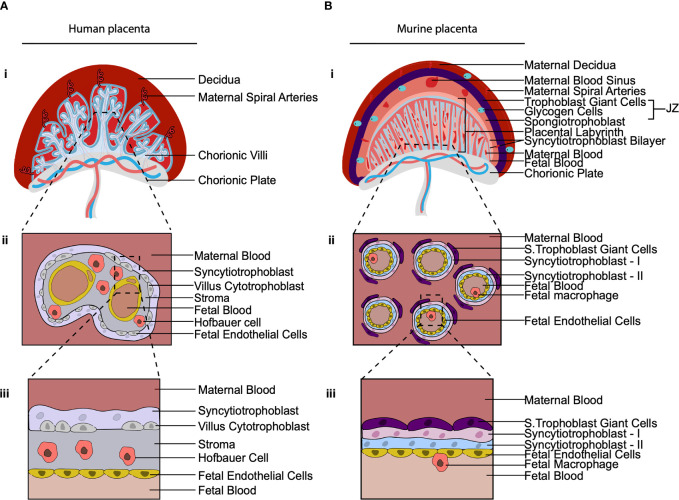
The cellular composition of the human and murine placenta. **(A, B)** Illustration of 2^nd^ trimester human placenta **(A)** and murine placenta **(B)**. (i) Both have a discoid shape and are hemochorial (bathed in maternal blood). (ii) Cross section of the placental villus region. The human placenta is hemo(mono)chorial (one layer of trophoblast separates fetal and maternal blood). The murine placenta is hemo(tri)chorial (three layers of trophoblast separate fetal and maternal blood; syncytiotrophoblasts-I, syncytiotrophoblasts-II and sinusoidal trophoblast giant cells). (iii) Close up of the hemochorial barrier separating fetal and maternal blood. In the human placental HBC are found within the stroma between trophoblast and fetal endothelial. In the murine placenta, macrophages have been found in the placental blood vessels. JZ, Junctional Zone; S. Trophoblast Giant Cells, Sinusoidal Trophoblast Giant cells.

Across species placentas vary in structure, cellular subtypes and the extent to which the placenta mediates fetal-maternal exchange (74). The structure of the murine placenta, for example, is similar to the human as it is discoid in shape and hemochorial, meaning the fetal trophoblast cells are directly bathed in maternal blood (75) (**Figure 3**). However, there are a number of differences between the murine and human placenta, that are excellently reviewed elsewhere (76). Of relevance here are differences between murine and human placental macrophages. Murine placental macrophages have been proposed to be analogous to human HBC, hence they have been termed HBC-like cells (77). However, murine placental macrophages that have been characterized thus far are not like human HBC in terms of ontogeny and localization. Human HBC first appear at day 18 post conception, when primitive hematopoiesis is still ongoing. In contrast, murine placental macrophages that have been identified, emerge from the placental vasculature at E10 HSC (78), coinciding with when definitive hematopoiesis has also begun in the murine AGM. The timing of their appearance suggests that human and murine fetal placental macrophages are derived from distinct waves of hematopoiesis, however, this has yet to be confirmed *via* fate mapping of murine placental macrophages. That is, human HBC are derived from primitive HSC while murine labyrinth macrophages are derived from definitive HSC. In terms of localization, the murine placental labyrinth has a greatly reduced to no interstitial space between the trophoblast layers and fetal endothelial cells in comparison with the human placenta (**Figure 3**). Murine labyrinth macrophages are primarily located within placental blood vessels (78). This is in stark contrast to human HBC that are found in abundance in the interstitial space between the trophoblast cells and the fetal endothelial cells. The highly divergent physical niches in which these cells reside strongly implies that murine and human placental macrophages have distinct functional roles. Due to these differences in ontogeny, localization, and likely function, we suggest that murine labyrinth macrophages should not be termed HBC-like cells.

In other species, such as non-human primates (79) and sheep (80), HBC-like cells have been found within the interstitial space between the trophoblast cells and the fetal endothelial cells of the placental villi. However, these macrophage populations remain poorly described. Due to the lack of an easily manipulatable animal model to study HBC, human placental samples remain the best resource for studying this cell type. To overcome the inherent limitations of working with human samples a number of approaches can be taken.

We now possess the means of isolating viable HBC and PAMM1a with a high degree of accuracy and precision for *in vitro* functional assays (3, 81). Profiling the responses of placental macrophages to a wider range of pathogens *in vitro* should help provide further mechanistic insights into the basis of transplacental infections. Placental explant cultures have been used in a number of studies to provide an experimental model for placental function in response to damage (34) and infection (49). These models are an attractive prospect for studying placental macrophage function, however there are issues relating to cell viability (82). A consistent problem with working with primary human fetal samples is the scarcity of samples. To maximize the output from these rare samples, studies often employ high-dimensional techniques. Recently the placenta has been profiled at both the first trimester and full term by scRNAseq (35, 83–85), which has provided significant insight into the properties of placental macrophages in homeostasis. Coupling these techniques with new methods to profile spatial transcriptomics from tissue sections (86, 87) will provide further insight into the local cell-cell communication networks which govern placental macrophage function. However, the combination of these techniques with either primary samples from pathological pregnancies, or with *in vitro* infected placental macrophages and whole explants are likely to provide the most significant advances in the field of placental macrophage research in the future. Using these high-dimensional methods to understand how both HBC and PAMM1a phenotypes, transcriptomes and metabolism vary under different conditions will help to develop our understanding of the roles of these cells in homeostasis and disease.

## Conclusions and Perspectives

With newly emerging pathogens it is important that we continue to develop tools to understand the mechanisms the placenta has in place to protect it from disease. The ability to rapidly determine if newly emerging microbes are a risk to pregnant women and their offspring is essential. HBC and PAMM1a are likely to be crucial components in the defense of the fetus against infection, as well as the normal function of the placenta. A caveat of furthering our understanding of HBC and PAMM1a is the lack of suitable models to study these cells. Without the ability to design an experiment that can manipulate their properties *in vivo*, it is difficult to determine the essential role of HBC and PAMM1a. However, the recent development of protocols that allow the study of primary human placental cells *in vitro*, will allow us to rapidly develop our understanding of these cells in both health and disease.

## Author Contributions

NM and JT drafted the manuscript. All authors provided scientific insight. Figures were created by JT and PN. All authors contributed to the article and approved the submitted version.

## Funding

NM is funded by a Wellcome Sir Henry Dale and Royal Society Fellowship (grant number 204464/Z/16/Z). JT is funded by a Wellcome Trust PhD Studentship (grant number 215226/Z/19/Z).

## Conflict of Interest

The authors declare that the research was conducted in the absence of any commercial or financial relationships that could be construed as a potential conflict of interest.

## Publisher’s Note

All claims expressed in this article are solely those of the authors and do not necessarily represent those of their affiliated organizations, or those of the publisher, the editors and the reviewers. Any product that may be evaluated in this article, or claim that may be made by its manufacturer, is not guaranteed or endorsed by the publisher.
